# Serious Adverse Events Are Uncommon with Combination Neonatal Antiretroviral Prophylaxis: A Retrospective Case Review

**DOI:** 10.1371/journal.pone.0127062

**Published:** 2015-05-22

**Authors:** Christiana Smith, Jeri E. Forster, Myron J. Levin, Jill Davies, Jennifer Pappas, Kay Kinzie, Emily Barr, Suzanne Paul, Elizabeth J. McFarland, Adriana Weinberg

**Affiliations:** 1 Department of Pediatric Infectious Diseases, University of Colorado School of Medicine and Children’s Hospital Colorado, Aurora, Colorado, United States of America; 2 Department of Biostatistics and Informatics, Colorado School of Public Health, Aurora, Colorado, United States of America; 3 Department of Medicine, University of Colorado School of Medicine, Aurora, Colorado, United States of America; 4 Department of Obstetrics and Gynecology, University of Colorado School of Medicine, Aurora, Colorado, United States of America; 5 Department of Obstetrics and Gynecology, Denver Health Medical Center, Denver, Colorado, United States of America; 6 Children's Hospital Colorado, Aurora, Colorado, United States of America; 7 Department of Pathology, University of Colorado School of Medicine, Aurora, Colorado, United States of America; University of British Columbia, CANADA

## Abstract

Six weeks of zidovudine (ZDV) is recommended for postnatal prophylaxis of HIV-exposed infants, but combination antiretrovirals are indicated if HIV transmission risk is increased. We investigated the frequency and severity of adverse events (AE) in infants receiving multiple drug prophylaxis compared to ZDV alone. In this retrospective review of 148 HIV-exposed uninfected infants born between 1997–2009, we determined clinical and laboratory AE that occurred between days of life 8–42. Thirty-six infants received combination prophylaxis; among those, a three-drug regimen containing ZDV, lamivudine, and nevirapine was most common (53%). Rates of laboratory AE grade ≥1 were as follows for the combination prophylaxis and ZDV alone groups, respectively: neutropenia 55% and 39%; anemia 50% and 39%; thrombocytopenia 0 and 3%; elevated aspartate aminotransferase 3% and 3%; elevated alanine aminotransferase 0 and 1%; hyperbilirubinemia 19% and 42%. Anemia occurred more frequently in infants who received three-drug prophylaxis compared to infants who received ZDV alone (63% vs. 39%, p = 0.04); all anemia AE were grade 1 or 2 in the three-drug prophylaxis group. Overall, 75% of infants on combination prophylaxis and 66% of infants on ZDV alone developed grade ≥1 AE (p = 0.32), and 17% of infants in either group developed grade ≥3 AE. Stavudine was substituted for ZDV in 23 infants due to anemia or neutropenia. After this antiretroviral change, 50% of evaluable infants demonstrated improvement in AE grade, and 25% had no change. In conclusion, low grade anemia, neutropenia, and hyperbilirubinemia occurred frequently regardless of the prophylactic regimen, but serious AE were uncommon. Although most AE were typical of ZDV toxicity, the combination of ZDV with lamivudine and nevirapine resulted in an increased frequency of low-grade anemia. Further studies are needed to identify prophylactic regimens with less toxicity for infants born to HIV-infected mothers.

## Introduction

Perinatal transmission of HIV has been reduced to less than 1% with use of antenatal, intrapartum, and postnatal antiretroviral prophylaxis (ARV) [1, 2]. In the U.S., standard postnatal prophylaxis for infants born to mothers with well-controlled HIV consists of six weeks of zidovudine (ZDV). For infants with increased transmission risk, such as those with absent or incomplete antenatal prophylaxis, or elevated maternal viral load at delivery, ZDV with the addition of three doses of nevirapine (NVP) in the first week of life is recommended [3]. However, the use of three-drug postnatal prophylaxis has increased substantially over the last decade in the U.S. and Europe [4–6]. In addition, administration of combination ARV soon after birth to infants at high risk of perinatal transmission has been proposed as a mechanism of limiting the latent viral reservoir in infants who become infected [7]. However, safety data are lacking for infants who receive combination ARV in the early postnatal period, particularly for three-drug regimens. A prior study comparing infants treated with ZDV plus lamivudine (3TC) vs. ZDV alone found no difference in the frequency of moderate-to-severe anemia and neutropenia at age 1 month [8]. In contrast, the recent NICHD-HPTN 040/PACTG 1043 trial, which compared three postnatal prophylaxis regimens in infants who did not receive antenatal prophylaxis, found more neutropenia in infants who received ZDV, 3TC, and nelfinavir than in infants who received ZDV and three doses of NVP, or infants who received ZDV alone [9]. Here we report a low incidence of severe adverse events (AE) in HIV-exposed, uninfected infants who received either combination postnatal ARV or ZDV alone.

## Methods

### Study Design

This study was reviewed and approved by the Colorado Multiple Institutional Review Board and exempted from informed consent on December 7, 2004. The data were analyzed anonymously and thus informed consent was not required. We reviewed medical records for all pregnancies complicated by HIV that were managed by the Children’s Hospital Immunodeficiency Program (CHIP) from 1997 to 2009. CHIP is the reference center for the care of HIV-infected pregnant women in Colorado and neighboring states. Clinical data were collected via chart abstraction, including mode of delivery (vaginal vs. Cesarean section), gestational age at delivery (preterm defined as <37 weeks), Apgar scores, birth weight, antenatal and postnatal ARV, infant laboratory values [hemoglobin, absolute neutrophil count, platelet count, aspartate aminotransferase (AST), alanine aminotransferase (ALT), and total bilirubin], and hospitalizations or illnesses during days of life (DOL) 8–42. Standard infant laboratory monitoring at this site included a complete blood count and liver function panel at birth, four weeks, and six weeks. Laboratory data obtained prior to DOL 8 were not included in the analysis, as too little time had passed for postnatal ARV to affect infant laboratory results. Infants with no laboratory values between DOL 8–42 were excluded.

Adverse events were graded using the Division of AIDS Table for Grading the Severity of Adult and Pediatric Adverse Events, dated December, 2004 [10]. If the medical record did not specify an upper limit of normal for AST and ALT, the limits imposed were 60 IU/L and 65 IU/L for AST and ALT, respectively, as these are the limits used by the Children’s Hospital Colorado laboratory. Infant HIV infection status was monitored using HIV RNA and/or DNA PCR at birth, 2 weeks, 4 weeks, 6 weeks, and 4 months of age, and HIV antibody starting at 12 months of age and repeated every 3–6 months until seroreversion was demonstrated.

### Antiretroviral prophylaxis

Mothers were routinely treated with highly active antiretroviral therapy (HAART; ≥3 antiretrovirals from ≥2 antiretroviral classes); modifications during the pregnancy were based on drug levels, virologic response, safety and tolerability, as described previously [11–13]. Most infants were prescribed postnatal prophylaxis consisting of six weeks of ZDV. In some infants, ZDV was replaced by stavudine (d4T) due to anemia, neutropenia, or both. In situations where the risk of perinatal transmission was increased, infants received two- or three-drug ARV. The second and/or third drug was typically prescribed for four weeks, based on adult post-exposure prophylaxis guidelines [14], but ZDV or d4T was continued for six weeks to comply with national recommendations for prevention of perinatal transmission [3]. Zidovudine and single dose NVP were dosed according to published guidelines available during the birth year. When used daily, NVP was dosed at 2mg/kg once daily for 1–2 weeks and then twice daily for the remainder of the course. Lamivudine was dosed at 2mg/kg twice daily. Complete dosing information is detailed in S1 Table. Decisions regarding infant ARV were made on a case-by-case basis by the CHIP perinatal clinicians. Trimethoprim/sulfamethoxazole was administered as prophylaxis for *Pneumocystis jiroveci* pneumonia to 39% of infants who received combination ARV, and 27% of infants who received ZDV alone; however, it was typically initiated during or after six weeks of life, outside of the interval assessed in this study.

### Statistical Analysis

Logistic regression was used to model grade ≥1 AE and grade ≥3 AE (yes/no) as a function of the type of postnatal prophylaxis received. The highest grade laboratory AE for each infant that occurred between DOL 8–42 was compared between those who received ZDV alone, combination ARV, and three-drug ARV, using logistic regression, chi-square tests or Fisher’s exact tests, as appropriate. Statistical significance was defined by p<0.05.

## Results

Between 1997 and 2009, 165 mothers were managed at CHIP for 190 pregnancies. One hundred and forty-eight infants were included in this analysis after exclusion of 42 infants without laboratory data between DOL 8–42. All infants were uninfected. Ninety percent of mothers received HAART. One hundred and twelve infants (including 4 sets of twins) received ZDV alone, and 36 infants received combination ARV. The infant demographics and maternal HIV characteristics are reported in Table 1. Baseline characteristics were similar between groups except for maternal viral load and CD4 T-lymphocyte count, reflecting more advanced maternal HIV disease in the combination ARV group. The proportion of infants born preterm, a known risk factor for AE, was not significantly different for those receiving combination ARV compared to ZDV alone [9/36 (25%) vs. 17/112 (15%), p = 0.21].

**Table 1:** Infant demographics and maternal HIV characteristics.

Characteristic	Number of infants/Number evaluated (%)	
	Zidovudine alone	Combination prophylaxis
Race^a^		
White	63/112 (56%)	16/36 (44%)
African American	38/112 (34%)	14/36 (39%)
American Indian/Alaskan Native	1/112 (1%)	1/36 (3%)
Other/Unknown	10/112 (9%)	5/36 (14%)
Ethnicity^a^		
Hispanic	42/112 (38%)	9/36 (25%)
Not Hispanic	59/112 (53%)	22/36 (61%)
Other/Unknown	11/112 (10%)	5/36 (14%)
Infant sex, male	59/110 (54%)	18/36 (50%)
Gestational age at delivery in weeks, mean (range)	37.7 (25–41), n = 109	37.0 (27–42), n = 33
Initial maternal viral load (copies/mL)^b^, median (range)	817 (<20–140 000), n = 106	6505 (<20–213 191), n = 32
Latest maternal viral load (copies/mL)^c^, median (range)	<48 (<20–6180), n = 60	685 (<20–86 838), n = 23
Initial maternal CD4 count^b^		
<200 cells/mm^3^	7/103 (7%)	4/28 (14%)
200–500 cells/mm^3^	39/103 (38%)	16/28 (57%)
>500 cells/mm^3^	57/103 (55%)	8/28 (29%)

^a^ Infant race and ethnicity determined by maternal self-report.

^b^ From earliest known maternal laboratory values during pregnancy.

^c^ From last known maternal laboratory values within 28 days prior to and including the date of delivery.

The combination ARV regimens administered are described in Table 2; additional detail is provided in S1 Table. Among the 36 infants receiving combination ARV, the majority (55%) received three-drug prophylaxis for ≥2 weeks, most commonly ZDV, 3TC, and NVP (53%). One infant received three-drug prophylaxis that included lopinavir/ritonavir. Three infants (8%) received two nucleoside reverse transcriptase inhibitors (NRTI) plus one or two doses of NVP, six infants (17%) received two NRTI, and four infants (11%) received ZDV plus single-dose NVP. The most common reasons for initiation of combination ARV included detectable maternal viral load at delivery (53%), inadequate antenatal prophylaxis (31%), prolonged rupture of membranes (25%), and exposure to excess maternal blood (17%).

**Table 2:** Infant prophylaxis.

Prophylactic antiretrovirals	No. of infants	Stavudine substituted for zidovudine^a^
zidovudine^b^ + lamivudine and nevirapine (≥2 weeks)^c^	19	8
zidovudine^b^ + lamivudine and lopinavir/ritonavir (4 weeks)^d^	1	1
zidovudine^b^ + lamivudine (2 weeks) + single dose nevirapine	2	0
stavudine^e^ and lamivudine (7 weeks) + two single doses nevirapine	1	0
zidovudine^b^ + lamivudine (4 weeks)	6	2
zidovudine^b^ + single dose nevirapine	4	0
zidovudine^b^ + other antiretroviral(s) (1–2 days)	3	0
zidovudine^b^ alone	112	12

^a^ The substitution of stavudine occurred at a median age of 30 days (range, 8–40 days)

^b^ Zidovudine was administered for at least 6 weeks in all infants.

^c^ One infant also received 1 week of ritonavir.

^d^ Treatment prescribed prior to warnings from the Food and Drug Administration against use of ritonavir-boosted lopinavir in infants younger than age 14 days.

^e^ This infant received stavudine rather than zidovudine due to maternal receipt of stavudine antenatally.

**Fig 1:**
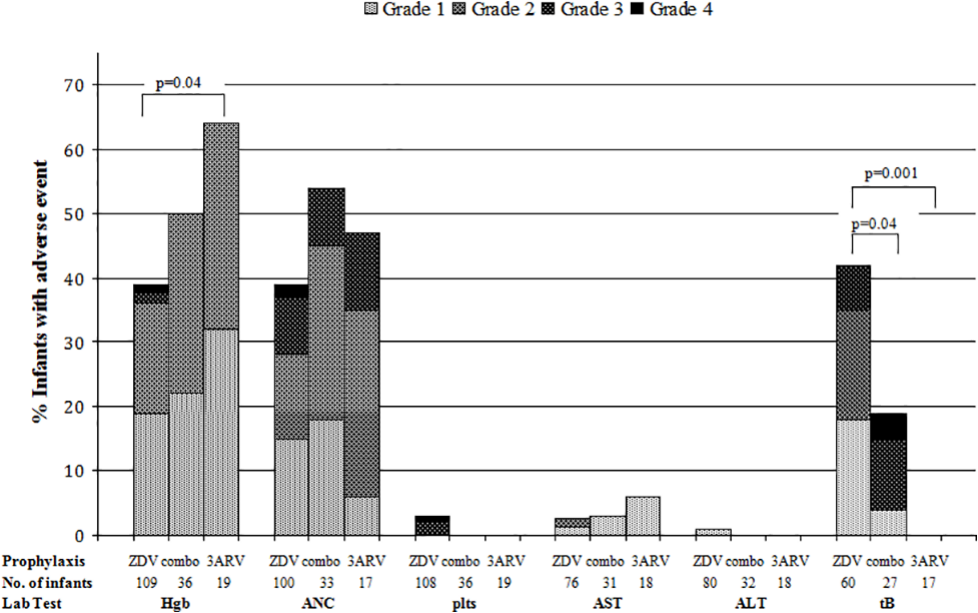
Frequency and severity of laboratory adverse events for infants exposed to zidovudine alone, combination prophylaxis, or three-drug prophylaxis. The maximum grade adverse event within each laboratory test (hemoglobin, Hgb; absolute neutrophil count, ANC; platelets, plts; aspartate aminotransferase, AST; alanine aminotransferase, ALT; total bilirubin, tB) that occurred between days of life 8 through 42 is shown for infants exposed postnatally to zidovudine alone (ZDV), combination antiretroviral prophylaxis (combo), and three-drug prophylaxis containing zidovudine (or stavudine), lamivudine, and nevirapine (3ARV). Significant differences are denoted by p-values.

The mean number of laboratory tests per subject performed in each group was similar: 1.36 vs. 1.11 complete blood counts and 1.12 vs. 1.01 liver function tests were performed in the combination ARV and ZDV alone groups, respectively. Laboratory AE experienced by both groups are shown in Fig 1. Anemia grade ≥1 occurred in 50% vs. 39%, and grade ≥3 occurred in 0 vs. 3%, of infants receiving combination ARV vs. ZDV alone, respectively. Neutropenia grade ≥1 occurred in 55% vs. 39%, and grade ≥3 occurred in 9% vs. 11%, respectively. Hyperbilirubinemia grade ≥1 occurred in 19% vs. 42% (p = 0.04), and grade ≥3 occurred in 15% vs. 7%, respectively. Thrombocytopenia grade ≥1 (0 vs. 3%), elevated AST grade ≥1 (3% vs. 3%), and elevated ALT grade ≥1 (0 vs. 1%) were uncommon. Except for hyperbilirubinemia, there were no significant differences in the frequency of AE within each laboratory category, between infants receiving combination ARV vs. ZDV alone. One infant, who was born preterm at 28 weeks’ gestation and received ZDV alone, required blood transfusions on DOL 16 and 17 due to severe anemia (nadir hematocrit of 29%; grade 3). One infant who received three-drug prophylaxis with ZDV, 3TC, and NVP, discontinued NVP early due to grade 1 elevated AST.

**Fig 2:**
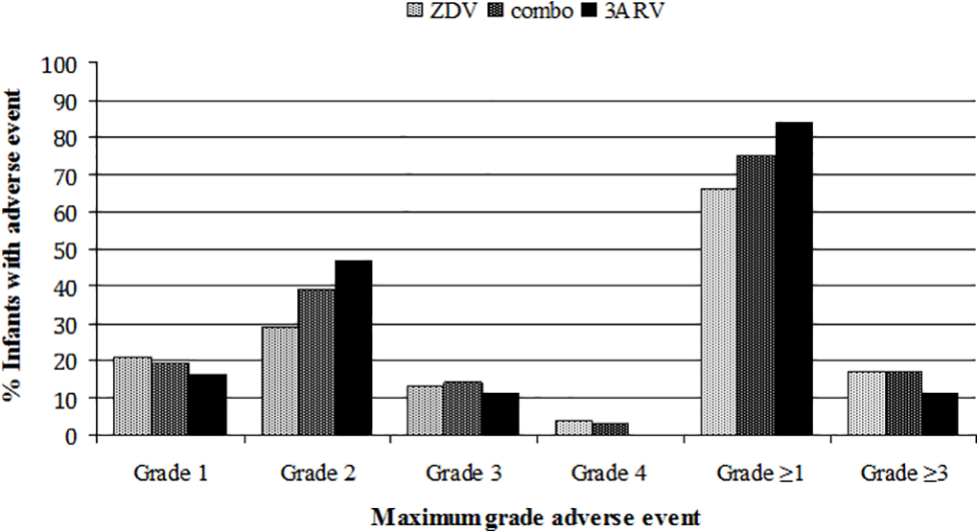
Comparison of highest grade adverse event between infants exposed to zidovudine alone, combination prophylaxis, or three-drug prophylaxis. The proportion of infants having an adverse event of the specified grade is determined using each infant’s highest grade adverse event between days of life 8 through 42 for any of the six laboratory tests evaluated (hemoglobin, absolute neutrophil count, platelet count, aspartate aminotransferase, alanine aminotransferase, total bilirubin). No significant differences were found between infants exposed to zidovudine alone (ZDV), combination antiretroviral prophylaxis (combo), and three-drug prophylaxis containing zidovudine (or stavudine), lamivudine, and nevirapine (3ARV).

Although low grade laboratory AE were common in both groups, there was no significant difference in the overall severity of AE for infants receiving combination ARV compared with those receiving ZDV alone. When comparing the highest grade AE across all six laboratory tests, the proportion of infants with an AE grade ≥1 was 75% vs. 66% (p = 0.32) for combination ARV vs. ZDV alone, respectively, and the proportion of infants with an AE grade ≥3 was 17% in each treatment group (Fig 2).

Because the initiation of three-drug ARV regimens at birth in infants at high risk of perinatal infection has been proposed as an approach to limiting the latent HIV reservoir in those who are infected, we also evaluated AE in the 19 infants who received at least two weeks of a three-drug combination of ZDV (or d4T), 3TC, and NVP. Infants who received three-drug ARV developed anemia grade ≥1 more frequently than infants who received ZDV alone (63% vs. 39%, p = 0.04), but no infant in the three-drug group developed anemia grade ≥3 (Fig 1). The frequency of neutropenia grade ≥1 (47% vs. 39%) and elevated AST grade ≥1 (6% vs. 3%) was not significantly different from infants who received ZDV alone. Grade ≥3 AE only occurred for neutropenia in this group (12% vs. 11% in the ZDV alone group). No thrombocytopenia, elevated ALT, or hyperbilirubinemia AE developed in infants who received three-drug prophylaxis. The frequency of hyperbilirubinemia in this group was significantly lower than in infants who received ZDV alone (0 vs. 42%, p = 0.001). There was no significant difference in the overall severity of laboratory AE for infants receiving three-drug ARV compared to infants receiving ZDV alone: 84% vs. 66% (p = 0.11) developed an AE grade ≥1, and 11% vs. 17% (p = 0.74) developed an AE grade ≥3 (Fig 2).

Despite similar frequencies of AE, clinicians more often substituted d4T for ZDV in infants receiving combination ARV than in infants receiving ZDV alone (31% vs. 11%, p = 0.004). The indication most often cited was anemia (83%). Among all treatment groups, 12 of the 23 infants who were switched to d4T had follow up laboratory data available. Six of these infants (50%) demonstrated improvement in their anemia or neutropenia by at least one grade, and three infants (25%) demonstrated a stable AE grade. One infant showed worsening of the AE grade. Two infants (17%) showed improvement in anemia, but worsening of neutropenia. These outcomes could not be compared to those of the infants who continued ZDV, as there were too few follow up laboratory data available.

Clinical AE experienced during DOL 8–42 by infants receiving combination ARV and ZDV alone, respectively, included thrush [4 infants (11%); 10 infants (9%)], upper or lower respiratory tract infection [1 infant (3%); 3 infants (3%)], and candidal diaper rash [0 infants; 2 infants (2%)]. Eight infants (7%), all of whom received ZDV alone, had a single serious adverse event during DOL 8–42 (sepsis, aseptic meningitis, bacterial conjunctivitis, necrotizing enterocolitis, diarrhea, urinary tract infection, balanitis, and apneic event). Of the six infants with a proven or suspected bacterial infection, one infant had grade 1 neutropenia, three infants had no evidence of neutropenia, and two infants did not have a neutrophil count available during DOL 8–42.

## Discussion

Combination ARV prophylaxis did not appreciably increase the severity of infant AE compared with ZDV alone. Anemia occurred more frequently in infants who received three-drug prophylaxis with ZDV, 3TC, and NVP, but all of these AE were grade 1 or 2. We found a slightly higher frequency of grade ≥2 anemia in our three-drug prophylaxis group than was noted in the three-drug prophylaxis group in the NICHD-HPTN 040/PACTG 1043 trial (32% vs 26%), possibly owing to the fact that the mothers in that study did not receive antenatal ARV, which likely has a cumulative effect upon infant laboratory AE [9]. We did not find an increased frequency of neutropenia in infants who received three-drug prophylaxis, in contrast with the results of the NICHD-HPTN 040/PACTG 1043 trial. Our three-drug ARV regimen contains NVP rather than nelfinavir, which may have contributed to the differences in outcome.

Anemia, neutropenia, and hyperbilirubinemia occurred frequently in this study, both in infants receiving combination ARV and in those receiving ZDV alone, but most AE were not serious (82% were grade 1 or 2). Hyperbilirubinemia was not clinically relevant, as no infants required phototherapy or other treatment for hyperbilirubinemia between DOL 8–42. Clinically relevant hyperbilirubinemia typically develops within the first week of life [15]. In addition, pathologic hyperbilirubinemia has not been previously associated with the use of NRTI or NVP. The Division of AIDS table employs conservative grading criteria for bilirubin (a grade 1 AE is equal to 1.1–1.5 times the upper limit of normal, where the upper limit is 1.0 or 1.2 mg/dL for most laboratories [10], and many healthy infants would be expected to have bilirubin values >1.0 mg/dL during DOL 8–42 [15]); thus, much of the hyperbilirubinemia reported was likely physiologic.

We propose that most of the hematologic AE we observed were associated with the use of ZDV, both alone and as a component of combination ARV. This is supported by the similar incidence of hematologic AE in the combination and ZDV alone groups and by the fact that 75% of AE improved or stabilized after the substitution of d4T for ZDV. This finding argues for the identification of antiretrovirals safer than ZDV for postnatal prophylaxis. Among other NRTIs, abacavir has not been studied in infants and the risk of severe allergic reactions represents a considerable impediment. Tenofovir has been associated with bone mineralization deficits, which is particularly undesirable in a growing infant. Although prolonged use of d4T has been associated with mitochondrial toxicity and lipodystrophy in adults [16], and with lactic acidosis when used in combination with didanosine in pregnant women [17], pediatric clinical trials of d4T showed that it was relatively safe and well tolerated, and was associated with less hematologic toxicity than ZDV [18–21]. Our experience suggests that d4T may be a safe alternative to ZDV when used briefly for postnatal prophylaxis, and may produce less hematologic AE, although these findings should be interpreted with caution due to the small number of infants who received d4T in our study.

Infants who received combination ARV were more likely than infants receiving ZDV alone to undergo a change from ZDV to d4T. Although an increased frequency of grade 1 or 2 anemia occurred in infants who received the most complex combination regimen of ZDV, 3TC, and NVP, the decision to switch to d4T may have been confounded by a heightened level of concern of health care providers for the infants receiving combination ARV, which resulted in a lower threshold for changing ARV. This underscores the need for standard protocols for the substitution of ZDV or other ARV in neonates. We have since developed a standard operations procedure at our institution, whereby infants are switched from ZDV to d4T for hemoglobin ≤8.2 g/dL (grade 2) or absolute neutrophil count ≤500 cells/μL (grade 4) at four weeks of life.

We administered NVP as the non-NRTI component of combination ARV in most cases, as its safety and pharmacokinetics in infancy were well described at the time when ARV decisions were made [22, 23]. Intermittently dosed NVP in combination with six weeks of daily ZDV is currently recommended in the U.S. for infant postnatal prophylaxis in pregnancies lacking antenatal ARV (first dose of NVP within 48 hours of birth, second dose 48 hours after first dose, third dose 96 hours after second dose) [3]. Our data are the first to describe a cohort of infants who received NVP dosed once- or twice-daily as a component of three-drug postnatal prophylaxis.

Few data are available regarding the use of protease inhibitors in early infancy. Nelfinavir is inconsistently absorbed and is not currently recommended for infant prophylaxis [24]. Although lopinavir/ritonavir is safe for infants older than 14 days, its use prior to 14 days of life is contraindicated due to the risk of cardiac toxicity [25, 26]. There are few available data on alternative ARV that could be considered for combination postnatal prophylaxis regimens. For infants at high risk of HIV transmission, risks and benefits of combination ARV must be weighed.

The small number of clinical AE experienced by infants during DOL 8–42 precluded statistical comparisons between the combination ARV and ZDV alone groups. However, we did not note any obvious differences between the quality or quantity of AE reported.

We acknowledge several limitations in this study. The sample size was relatively small. Because this was a retrospective review, there were missing laboratory data, but proportions of missing data were similar between the combination ARV and ZDV alone groups. Infants were not randomized to particular prophylactic regimens; they received ARV based on the perceived risk of HIV transmission and using the knowledge of antiretroviral safety and tolerability at the time of each infant’s birth. Finally, the ARV regimens administered in the combination prophylaxis group were heterogeneous; however, we were able to describe AE in the group of infants who received a three-drug ARV regimen of ZDV (or d4T), 3TC, and NVP.

## Conclusions

We describe the incidence of AE in infants exposed to postnatal combination antiretroviral prophylaxis, a topic on which there are few previous data. We did not find a significant difference in the severity of AE between infants exposed to combination ARV vs. ZDV alone. These results must be interpreted with caution due to the relatively small number of infants who received combination ARV and the heterogeneity of the drug regimens administered. We did find an increased frequency of low-grade anemia in infants who received the most complex combination ARV regimen of ZDV, 3TC, and NVP. We also showed that d4T may be an acceptable NRTI for use in postnatal prophylaxis, especially if hematologic toxicity is a concern. Additional studies are needed of combination postnatal prophylaxis regimens, including the use of antiretrovirals other than ZDV.

## Supporting Information

S1 Table Detailed infant combination prophylaxis. A detailed description of the antiretroviral type, duration, year of administration, and indication is reported for each infant who received combination postnatal prophylaxis.
(DOCX)
