# Supplementary material for: Serious Adverse Events Are Uncommon with Combination Neonatal Antiretroviral Prophylaxis: A Retrospective Case Review
Source: PLoS One. 2015 May 22;10(5):e0127062. doi: 10.1371/journal.pone.0127062 (PMC4441417; doi:10.1371/journal.pone.0127062)
Supplement: S1 Table — A detailed description of the antiretroviral type, duration, year of administration, and indication is reported for each infant who received combination postnatal prophylaxis. (DOCX) [file pone.0127062.s001.docx]

**Table S1. Details of infant combination prophylaxis.**

| **Prophylactic antiretrovirals^a^** | **Duration**  **(weeks)** | **Birth Year** | **Indication** |
| --- | --- | --- | --- |
| **Three-drug regimens** | | | |
| zidovudine + lamivudine +nevirapine  zidovudine | 4  2 | 2009 | Inadequate antenatal prophylaxis |
| zidovudine +lamivudine + nevirapine  zidovudine | 2  4 | 2009 | Maternal viral load unknown^b^  Prolonged rupture of membranes |
| zidovudine + lamivudine + nevirapine  stavudine^c^ | 4  2 | 2009 | Detectable maternal viral load |
| zidovudine + lamivudine + nevirapine  stavudine^c^ | 4  2 | 2009 | Detectable maternal viral load Inadequate antenatal prophylaxis |
| zidovudine + lamivudine + nevirapine  zidovudine | 4  2 | 2008 | Inadequate antenatal prophylaxis |
| zidovudine + lamivudine + nevirapine  zidovudine | 4  2 | 2008 | Detectable maternal viral load Inadequate antenatal prophylaxis |
| zidovudine + lamivudine + nevirapine^d^  zidovudine + lamivudine  zidovudine | 3  1  2 | 2008 | Detectable maternal viral load  Prolonged rupture of membranes |
| zidovudine + lamivudine + nevirapine  stavudine^c^ | 4  2 | 2008 | No antenatal prophylaxis |
| zidovudine + lamivudine + nevirapine  stavudine^c^ + lamivudine + nevirapine  stavudine | 3  1  2 | 2008 | Detectable maternal viral load |
| zidovudine + lamivudine + nevirapine  zidovudine | 4  2 | 2007 | Detectable maternal viral load  Inadequate antenatal prophylaxis  Prolonged rupture of membranes |
| zidovudine  zidovudine + lamivudine + nevirapine^e^  stavudine^c^ + lamivudine + nevirapine  stavudine | 1  2  1  2 | 2007 | Prolonged rupture of membranes |
| zidovudine +lamivudine + lopinavir/ritonavir^f^  stavudine^c^ | 4  2 | 2007 | Inadequate antenatal prophylaxis |
| zidovudine + lamivudine + nevirapine  zidovudine  stavudine^c^ | 4  1.5  0.5 | 2006 | Inadequate antenatal prophylaxis |
| zidovudine + lamivudine + nevirapine  stavudine^c^ + lamivudine + nevirapine  stavudine | 1  3  2 | 2006 | No antenatal prophylaxis  Detectable maternal viral load  Invasive fetal monitoring device |
| zidovudine + lamivudine + nevirapine  zidovudine | 4  2 | 2005 | Exposure to excess maternal blood |
| zidovudine + lamivudine + ritonavir  zidovudine + lamivudine + nevirapine  stavudine^c^ | 1  3  2 | 2004 | Detectable maternal viral load |
| zidovudine + lamivudine + nevirapine  zidovudine | 4  2 | 2003 | Detectable maternal viral load Exposure to excess maternal blood |
| zidovudine + lamivudine  nevirapine  zidovudine + lamivudine + nevirapine^e^  zidovudine | 1  single dose  3  2 | 2002 | Detectable maternal viral load  No antenatal prophylaxis  Prolonged rupture of membranes |
| zidovudine + lamivudine  nevirapine  zidovudine + lamivudine + nevirapine  zidovudine | 1  2 single doses  4  1 | 2002 | Detectable maternal viral load |
| zidovudine + lamivudine  nevirapine  zidovudine + lamivudine + nevirapine  zidovudine | 1  single dose  3  2 | 2001 | Detectable maternal viral load Exposure to excess maternal blood |
| **Two nucleoside reverse transcriptase inhibitors plus single dose nevirapine** | | | |
| zidovudine + lamivudine  nevirapine  zidovudine | 2  single dose  4 | 2001 | Detectable maternal viral load |
| zidovudine + lamivudine  nevirapine  zidovudine | 2  single dose  4 | 2000 | Exposure to excess maternal blood  Prolonged rupture of membranes |
| stavudine^g^ + lamivudine  nevirapine | 7  2 single doses | 1999 | Inadequate antenatal prophylaxis |
| **Two nucleoside reverse transcriptase inhibitors** | | | |
| zidovudine + lamivudine  stavudine^c^ | 4  2 | 2009 | Prolonged rupture of membranes |
| zidovudine + lamivudine  zidovudine | 4  2 | 2008 | Detectable maternal viral load |
| zidovudine + lamivudine  zidovudine | 4  2 | 2008 | Invasive fetal monitoring device |
| zidovudine +lamivudine  stavudine^c^ +lamivudine  stavudine | 3  1  2 | 2008 | Detectable maternal viral load  Prolonged rupture of membranes |
| zidovudine + lamivudine  zidovudine | 4  2 | 2006 | Detectable maternal viral load |
| zidovudine (preterm infant dosing)  zidovudine + lamivudine  zidovudine | 2 days  4  2 | 2004 | Inadequate antenatal prophylaxis |
| **Zidovudine plus single dose nevirapine** | | | |
| zidovudine  nevirapine | 6  single dose | 2007 | Inadequate antenatal prophylaxis |
| zidovudine  nevirapine | 7  single dose | 2000 | Detectable maternal viral load |
| zidovudine  nevirapine | 6  single dose | 2000 | Detectable maternal viral load |
| zidovudine  nevirapine | 6  single dose | 1997 | ACTG 316^h^ |
| **Zidovudine plus other antiretroviral(s)** | | | |
| zidovudine  zidovudine + lamivudine + nevirapine^e,i^  zidovudine | 2  2 days  4 | 2009 | Detectable maternal viral load  Exposure to excess maternal blood  Prolonged rupture of membranes |
| zidovudine + lamivudine + nevirapine  zidovudine | 1 day  6 | 2006 | Inadequate antenatal prophylaxis  Maternal viral load unknown^b^ |
| zidovudine (preterm infant dosing)  ritonavir | 6  2 doses | 2001 | Exposure to excess maternal blood |

A detailed description of the antiretroviral type, duration, year of administration, and indication is reported for each infant who received combination postnatal prophylaxis.

**^a^** Unless otherwise specified, postnatal antiretroviral prophylaxis was dosed as follows: lamivudine, 2mg per kg body weight PO twice daily; lopinavir/ritonavir, 16mg per kg body weight PO twice daily; nevirapine, 2mg per kg body weight PO once daily for one or two weeks, then increased to 2mg per kg body weight PO twice daily for remainder of course; single dose nevirapine, 2mg per kg body weight PO administered in the first 72 hours of life, if a second dose was given, this was administered within the first seven days of life; ritonavir, 2mg per kg body weight PO twice daily; stavudine, 1mg per kg body weight PO twice daily; zidovudine; 2mg per kg body weight PO four times daily, or 4mg per kg body weight PO twice daily; zidovudine preterm infant dose, 1.5mg per kg body weight IV twice daily, increasing to 2mg per kg body weight PO twice daily once oral medications are tolerated, increased to 2mg per kg body weight PO every 8 hours at 2 weeks of age for infants born at 30-35 weeks gestation, and at 4 weeks of age for infants born at <30 weeks gestation, increased to term infant dosing at 37 weeks corrected gestational age.

^b^ Maternal viral load unknown at delivery, but subsequently found to be undetectable. Thus, infant’s lamivudine and nevirapine were discontinued prior to completing a 4 week course.

^c^ Stavudine was substituted for zidovudine prior to completing a 6 week course, due to infant anemia, neutropenia, or both.

^d^ Nevirapine was discontinued prior to completing a 4 week course, due to infant liver enzyme elevation.

^e^ Lamivudine and/or nevirapine were not initiated until infant’s initial outpatient follow up visit.

^f^ Treatment prescribed prior to warnings from the Food and Drug Administration against use of ritonavir-boosted lopinavir in infants younger than age 14 days.

^g^This infant received stavudine rather than zidovudine due to maternal receipt of stavudine antenatally.

^h^ Mother and infant were enrolled in AIDS Clinical Trials Group (ACTG) 316, a pharmacokinetics study of nevirapine vs. placebo for prevention of perinatal transmission of HIV.

^i^ Lamivudine and nevirapine were discontinued prior to completing a 4 week course, due to noncompliance.
